# Identification of electron transfer enzymes in *Thermoanaerobacterium saccharolyticum*

**DOI:** 10.1128/jb.00107-25

**Published:** 2025-06-06

**Authors:** João H. T. M. Fabri, Layse C. de Souza, Luana W. Bergamo, Lee R. Lynd, Daniel G. Olson

**Affiliations:** 1 Centro de Biologia Molecular e Engenharia Genética, Universidade Estadual de Campinas 28132, Campinas, State of São Paulo, Brazil; 2 Terragia Biofuel Incorporated, Hanover, New Hampshire, USA; 3 Thayer School of Engineering at Dartmouth College, Hanover, New Hampshire, USA; 4 Center for Bioenergy Innovation, Oak Ridge National Laboratory 6146, Oak Ridge, Tennessee, USA; University of Southern California, Los Angeles, California, USA

**Keywords:** electron transfer, redox balance, ferredoxin:NAD(P)H oxidoreductase, hydrogenases, anaerobic thermophiles

## Abstract

*Thermoanaerobacterium saccharolyticum* is a promising candidate for the production of biofuels from lignocellulosic sugars; however, the genes associated with electron transfer from ferredoxin are poorly characterized. In this work, we deleted several key electron transfer genes. We showed that the *tsac_1705* gene is not necessary for high-yield ethanol production, but that a set of four other genes (*nfnA*, *nfnB*, *hfsD*, and *hydA*) are necessary. We showed that the *nfnB* gene can function as a monofunctional (i.e., non-bifurcating) FNOR enzyme in the absence of *nfnA*. The phenotypes of the *hfsD*, *hydA*, and *hfsD hydA* double-deletion strains are consistent with their function via hydrogen cycling.

**IMPORTANCE:**

The improved understanding of electron transfer pathways in *T. saccharolyticum* will enable future efforts to transfer the robust ethanol production pathway from this microbe to other organisms, with potential implications for industrial biofuel production.

## INTRODUCTION

**Fig 1:**
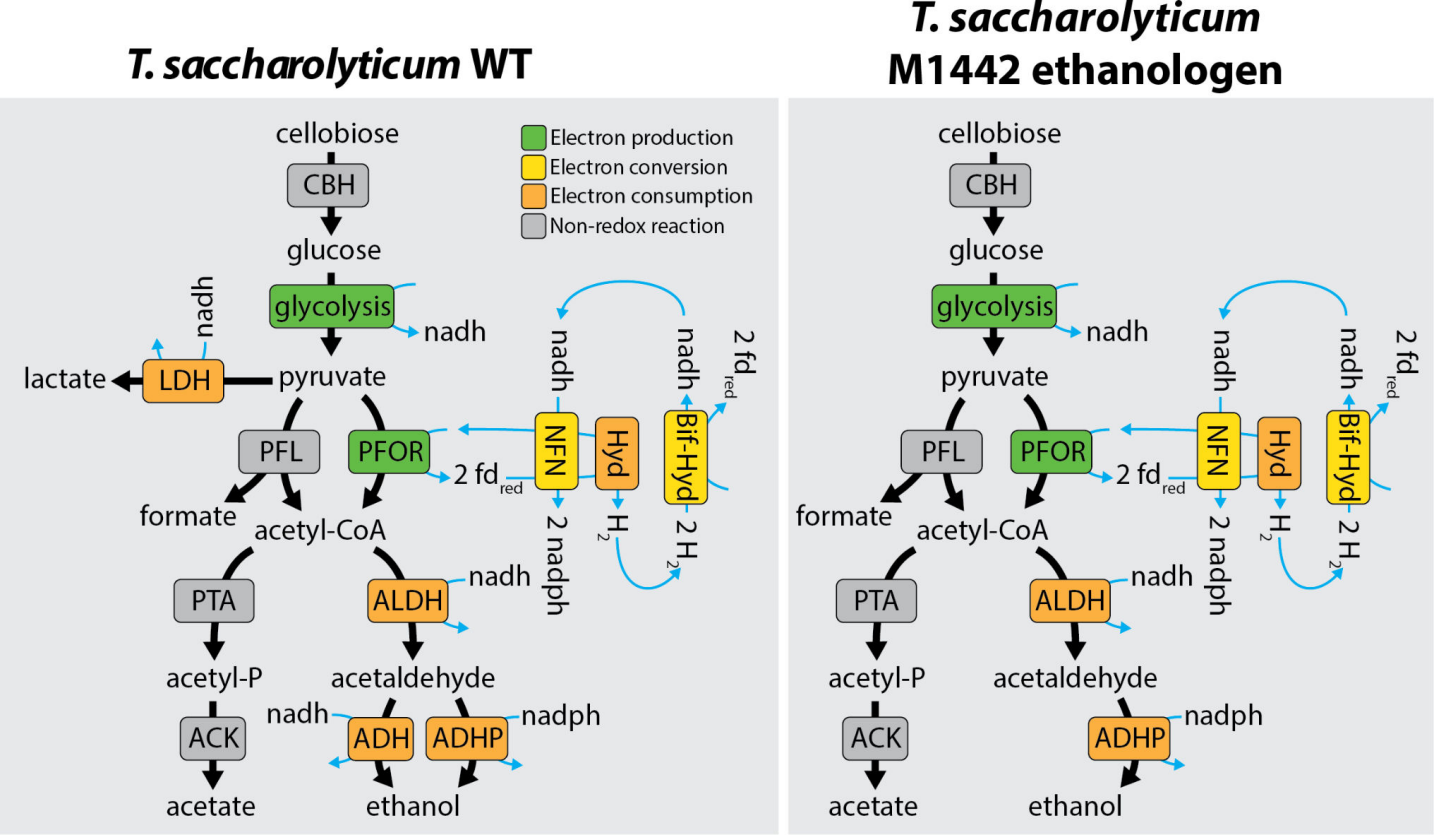
Metabolic diagram of key metabolic pathways in *T. saccharolyticum*, for both the WT (strain LL1025) and ethanologenic strains (M1442 and LL1328). Enzyme reactions are colored based on whether they generate reduced electron carriers (green), consume reduced electron carriers (orange), or interconvert between different types of reduced electron carriers (yellow). Black arrows show carbon conversions, and blue arrows show electron transfer. For redox cofactor pairs (NADH/NAD^+^, NADPH/NADP^+^, and Fd_red_/Fd_ox_) only the reduced form is shown, to avoid clutter. Abbreviations for enzymes include the following: cellobiose hydrolase (CBH), lactate dehydrogenase (LDH), pyruvate formate lyase (PFL), pyruvate ferredoxin oxidoreductase (PFOR), phosphotransacetylase (PTA), acetate kinase (ACK), acetaldehyde dehydrogenase (ALDH), alcohol dehydrogenase (ADH), NADPH-linked alcohol dehydrogenase (ADHP), NADH-dependent reduced ferredoxin: NADP^+^ reductase (NFN), electron bifurcating hydrogenase (Bif-Hyd), and ferredoxin hydrogenase (Hyd). The 10 reactions of canonical EMP glycolysis are lumped together in a single “glycolysis” reaction.

*T. saccharolyticum* is an anaerobic thermophilic bacterium that has the potential to be used for conversion of lignocellulose-derived sugars into biofuels (1, 2). In addition, this organism provides an interesting example of ethanol production via a metabolic pathway that uses the pyruvate-ferredoxin oxidoreductase enzyme, rather than the more commonly used pyruvate decarboxylase enzyme (3). Ethanol production at titers of 70 g/L has been reported for engineered strains of this microorganism (1). It is stoichiometrically possible to produce one mol of ethanol per half mol of C6 sugar (1/2 C_6_H_12_O_6_ → 1 C_2_H_6_O + 1 CO_2_) at 97% thermodynamic efficiency (Fig. 1). To achieve this conversion, all of the electrons initially present in the C6 sugar must be transferred to ethanol and not diverted to organic acids or H_2_.

Electron transfer is an essential step in the ethanol production pathway of anaerobic thermophiles. Ferredoxins are iron-sulfur proteins found in many anaerobic bacteria mediating electron transfer in various metabolic processes, including alcohol production (4). In the ethanol production pathway of *T. saccharolyticum*, the pyruvate oxidation to acetyl-CoA is possible through electron transfer from ferredoxin to nicotinamide cofactors, which is catalyzed by ferredoxin:NAD(P)H oxidoreductases (known as FNOR enzymes) (5). These enzymes form a key bridge in metabolism between the nicotinamide cofactor (i.e., NAD^+^, NADH, NADP^+,^ and NADPH)-dependent pathways and ferredoxin-dependent pathways. Previously, our group had studied two different possible FNORs. The NfnAB enzyme complex is a bifunctional enzyme that allows the following NFN reaction (Equation 1) (6, 7):


(Eq. 1) $$2\;Fd_{red}^{1-}+2\;NADP^{+}+NADH+H^{+}\overset{}{\to }2\;Fd_{ox}+2\;NADPH+NAD^{+}$$

However, the NFN reaction alone is not sufficient to explain the high-yield ethanol production in this organism. First, strains of this organism lacking NfnAB have been developed, but those still produce ethanol at >50% of the theoretical maximum yield (and in fact, above 80%) (6) that implies electron transfer from reduced ferredoxin to NAD^+^. Second, the nicotinamide cofactor stoichiometry of the NFN reaction (2 NADPH produced per mole of pyruvate converted to acetyl-CoA) is not compatible with the nicotinamide cofactor usage of any of the known ethanol production pathways in *T. saccharolyticum* (either 1 NADH and 1 NADPH or 2 NADH) (6).

Since *T. saccharolyticum* does not include the proton-translocating ferredoxin:NAD + oxidoreductase (Rnf) enzyme, our initial search for this missing activity led us to the monofunctional Tsac_1705 enzyme, which we suggested allows the following reaction (Equation 2) (8):


(Eq. 2) $$2\;Fd_{red}^{1-}+NAD^{+}+H^{+}\overset{}{\to }2\;Fd_{ox}+NADH$$

However, its relatively low activity and our inability to transfer this pathway to other organisms made us reconsider the role of Tsac_1705 in *T. saccharolyticum* ethanol production. Furthermore, the recent suggestion that hydrogen cycling between hydrogenases could function as an electron transfer pathway in *Thermoanaerobacterium thermosaccharolyticum* (9) provided additional motivation to reconsider our understanding of electron transfer pathways in *T. saccharolyticum*.

Hydrogenases are integral components of electron recycling inside the cell by producing or uptaking molecular hydrogen. In *T. saccharolyticum*, three gene clusters are involved in hydrogenase activity: the *hyd*, *ech,* and *hfs* gene clusters (10). Recent work has shown that *hfsD*, along with *hydAB* from the *hyd* gene cluster, is necessary for high ethanol yields in *T. thermosaccharolyticum* (9). The orthologs of some of these hydrogenases in *Clostridium thermocellum* seem to act in the same step as the FNORs, i.e., in the oxidation of pyruvate to acetyl-CoA (11). This made us consider: could the hydrogenases act together with the FNOR enzymes to promote electron transfer?

Thus, we set out to answer the following questions: (i) How are electrons transferred from ferredoxin to nicotinamide cofactors? and (ii) What is the minimal set of genes necessary for electron transfer and high-yield ethanol production in our best engineered ethanologenic strain of *T. saccharolyticum*? We approached this task primarily using the tools of genetics and microbial physiology. Our results shed light on the importance of FNORs and hydrogenases for ethanol production in *T. saccharolyticum*.

## MATERIALS AND METHODS

### Strain construction

The strains used in this study are listed in Table 1. Primers used for the construction of the strains and confirmation of deletions are listed in Table S1. Kanamycin marker deletions were made by transforming *T. saccharolyticum* cells with deletion cassettes consisting of a kanamycin (*kan*) marker and the thymidine kinase (*tdk*) gene flanked by upstream and downstream homology regions of the target gene (*nfnA*, *nfnB*, *hydA*, *hfsD*, and *tsac_1705*). The cassettes were constructed from linear PCR products via Gibson assembly (12), followed by anaerobic incubation with cells at 55°C to allow the naturally competent *T. saccharolyticum* cells to take up the DNA constructs (13). The transformants were selected in plates supplemented with 200 µg/mL kanamycin. For a second round of transformation, 5-fluoro-2’-deoxyuridine (FUDR) was used as the negative selection agent to remove the *kan-tdk* marker, as described previously (14).

**TABLE 1:** *T. saccharolyticum* strains used in this study^*a*^

Strain	Parent strain	Genotype	Source	Sequencing data
LL1025	–	Wild-type *T. saccharolyticum* JW/SL-YS485	(15)	GenBank accession no. CP003184.
M1442	LL1025	LL1025 Δ*pta* Δ*ack* Δl*dh adhE*^G544D^	Also called LL1049 (1)	SRA accession no. SRA233073.
LL1328	M1442	M1442 Δ*tdk*	(16)	–
LL1145	M0353	M0353 Δ*nfnAB*::kan	(6)	–
A2G0015	LL1328	M1442 Δ*tdk*Δ*tsac_1705::kan::tdk*	This work	SRA accession no. SRR32258721.
A2G0016	LL1328	M1442 Δ*tdk*Δ*nfnAB::kan::tdk*	This work	SRA accession no. SRR32258720.
A2G0017	LL1328	M1442 Δ*tdk*Δ*nfnB::kan::tdk*	This work	SRA accession no. SRR32258719.
A2G0018	LL1328	M1442 Δ*tdk*Δ*nfnA::kan::tdk*	This work	SRA accession no. SRR32258718.
A2G0019	LL1328	M1442 Δ*tdk* Δ*hydA::kan::tdk*	This work	SRA accession no. SRR32258717.
A2G0020	LL1328	M1442 Δ*tdk* Δ*hfsD::kan::tdk*	This work	SRA accession no. SRR32258716.
A2G0021	LL1328	M1442 Δ*tdk* Δ*hydA::*Δ*hfsD::kan::tdk*	This work	SRA accession no. SRR32258715.

^*a*^ – : not applicable or available.

### Media and growth conditions

All strains were grown anaerobically at 55°C. M122C rich medium at pH 6.5 was used for *T. saccharolyticum* transformation (17). For end-product analysis, *T. saccharolyticum* was grown in chemically defined MTC-6 medium (pH 6.5) (18). The MTC-6 medium was prepared as previously described (18), using 5 g/L or 20 g/L of cellobiose, depending on the experiment. For fermentation, an inoculum with 1% frozen cell culture was prepared in tubes containing 20 mL of medium, and these were incubated without shaking for 120 h or 240 h at 55°C. For gas chromatography analysis, fermentation was done in 125 mL glass bottles containing 25 mL of the medium incubated in an orbital shaking incubator for 240 h at 55°C and 200 rpm. To analyze the growth rates, cells were grown in 200 µL of the MTC-6 medium in a 96-well plate, and the absorbance at 600 nm was monitored every 8 min for 96 h.

### Fermentation end product analysis

Ethanol and other fermentation products in the liquid phase (cellobiose, lactate, acetate, and formate) were measured using a Shimadzu LC-2050C high-performance liquid chromatograph (HPLC) with an HPX-87H column and an additional UV detector (for the accurate quantification of pyruvate). The column was incubated at 60°C, and the mobile phase (H_2_SO_4_ 2.5 mM) flow rate was 0.6 mL/min. All fermentation data are reported in Table S2.

### Gas chromatography (GC) analysis

The concentration of hydrogen in the bottles after fermentation was analyzed using an SRI 310C gas chromatograph (SRI Inc.), utilizing a HayeSep D packed column and nitrogen as the carrier gas at a flow rate of 8.2 mL/min, as previously described (16). The oven temperature was maintained at 151°C and the current at 80 mA. The absolute H_2_ measurement was determined after correction for pressure differences. All gas chromatography data are reported in Table S3.

### Nuclear magnetic resonance (NMR) analysis

Measurement of additional fermentation products that were not detected by HPLC was done by nuclear magnetic resonance (NMR) analysis. To prepare the samples, 300 uL of each previously filtered fermentation sample was diluted in 240 uL of deuterium oxide and 60 uL of a 1 M phosphate buffer solution pH 7.4 containing 5 mM TMSP-d4 [3-(trimethylsilyl)propionic acid sodium salt]. The NMR experiment was carried out at LNBio (CNPEM), Campinas, Brazil. Briefly, 1H-NMR spectra were acquired using a Varian/Agilent DD2 spectrometer (Agilent Technologies Inc., Santa Clara, CA, USA) equipped with a triple-resonance probe and operating at a 1H resonance frequency of 499.72 MHz. Spectra acquisition was performed with 256 scans collected with 32 K data points over a spectral width of 8,000 Hz. A 2.0 s relaxation delay was incorporated between scans, during which a continual water pre-saturation radio frequency (RF) field was applied to eliminate the residual water signal.

The metabolites were processed and quantified using NMR Suite software version 8.1 (Chenomx Inc, Edmonton, AB, Canada). The processor module of this software was used to adjust the spectral phase and baseline corrections. A 0.3 Hz line-broadening function was used to reduce the signal noise and facilitate the fitting of the metabolite signals in spectral peaks. The water signal was suppressed, and the spectra were calibrated using the reference signal of the TMSP-d4 as 0.5 mM. The spectra were individually transferred to the Profiling module of this software to determine the metabolomic profile of each sample, as well as its concentration. All NMR data are reported in Table S4.

### Material balance calculations

Material balance calculations were performed by combining data from HPLC, GC, and NMR measurements. Endpoint metabolite concentration data were averaged per strain and then combined. Although our NMR and HPLC data showed similar trends, in cases where compounds were measured by both NMR and HPLC, we used the HPLC-generated data for material balance calculations. We consider the HPLC data to be more quantitatively accurate than the NMR data because each compound of the HPLC data was quantified with an individual external standard vs the NMR data that were quantified based on the single internal TMSP standard. For comparison of liquid-phase and gas-phase data, species were converted to units of millimoles (mmol) and normalized to a 20 mL fermentation volume. Available electron calculations are based on using coefficients of 4, 1,–2, and −3 available electrons per mole for the atoms C, H, O, and N, respectively (19). Estimated CO_2_ production was calculated based on assumptions of CO_2_ production for each compound [Table S5 - Compounds lookup from Material balance calculations.xlsx]. Raw data for all measurements are provided in Table S5.

### Whole-genome sequencing

The strains were grown in M122C rich medium, and the genomic DNA was extracted using the Monarch Genomic DNA Purification Kit (New England Biolabs). DNA quality was assessed using a TapeStation 4200 system (Agilent Technologies). The genomic DNA was submitted to the Life Sciences Core Facility (LaCTAD) from State University of Campinas (UNICAMP), where Illumina NextSeq2000 sequencing was performed. Paired-end reads were generated with an average read length of 150 bp, with a coverage of 5 million reads. Libraries were prepared using the Illumina DNA Prep Kit. Short reads were submitted to the NCBI’s Short Read Archive under the Bioproject PRJNA1220144. *De novo* genome assembly was done using the *T. saccharolyticum* JW/SL-YS485 genomic sequence (NCBI reference sequence NC_017992.1) as the reference in Geneious Prime 2024.0.5 software.

## RESULTS

### Effect of knockouts on growth, ethanol, and hydrogen production

**Fig 2:**
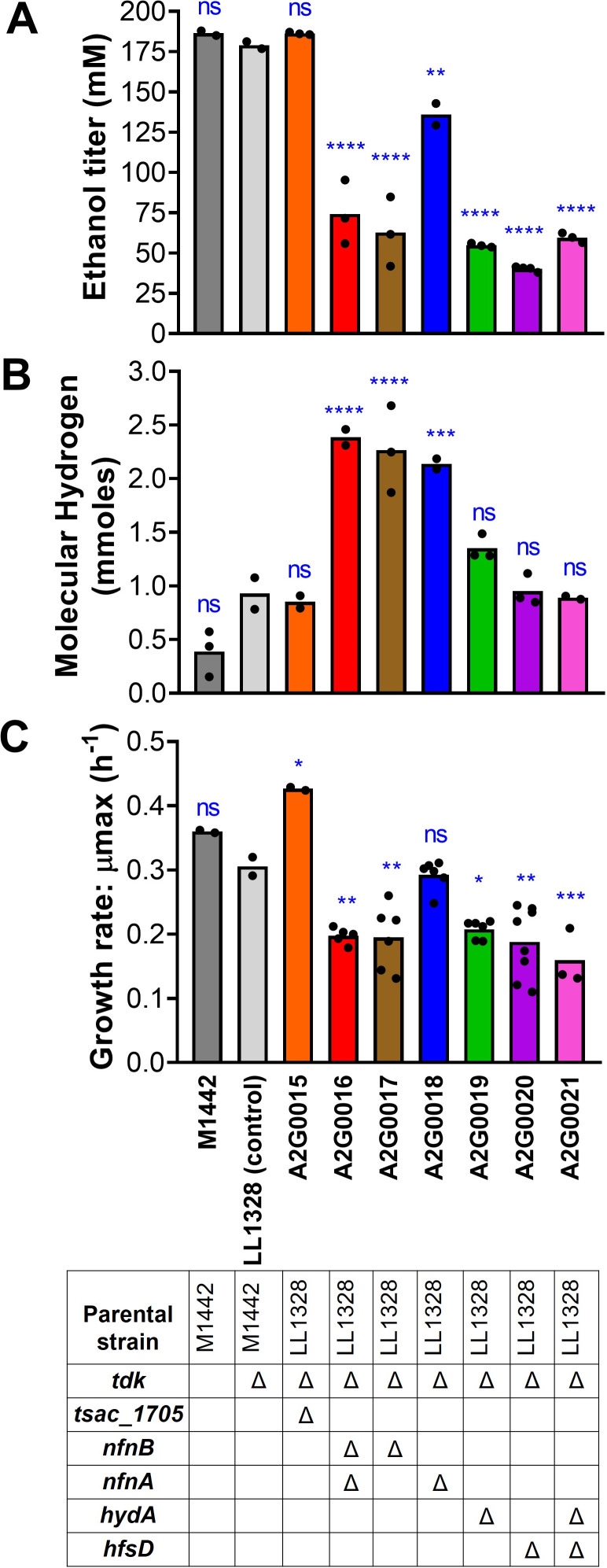
Ethanol titer, hydrogen production, and growth analysis of *T. saccharolyticum* knockout mutants. (A and B) All the strains were cultivated on the MTC-6 defined medium containing 20 g/L of cellobiose for 240 h. Black dots indicate the biological replicates. (**C**) Growth rates of the strains. For the growth analysis, the strains were cultivated in a 96-well plate containing the MTC-6 medium (5 g/L of cellobiose) for 96 h. Black dots indicate the biological replicates. * *P* ≤ 0.05, **P ≤ 0.01, ***P ≤ 0.001, and ****P ≤ 0.0001 (one-way ANOVA and Dunnett’s post-test in relation to LL1328 (control) strain—light gray bar). ns = non-significant. The genotype of each strain is shown in the table below the graphics, where blanks indicate the WT alleles and Δ indicates disruption of the gene by replacement with a *kan* marker. Full genotypes of the strains are listed in Table 1.

A key step in the ethanol production pathway of *T. saccharolyticum* is the transfer of electrons from ferredoxin to NAD^+^ and/or NADP^+^. Previously, we have seen that in the ethanologenic strain (strain ID M1442/LL1049), *nfnAB* deletion considerably decreased the ethanol yield, and the *nfnAB* knockout caused loss of NADPH-FNOR activity (6). Aiming to understand the individual participation of NfnA and NfnB in ethanol production and particularly whether NfnB can function independently of NfnA, both genes were individually and jointly deleted in the ethanologenic strain background. In this case, we used strain LL1328, derived from strain M1442 by the deletion of the *tdk* gene, which allows for subsequent removal of the antibiotic resistance marker if further genetic modifications are required (16). The resulting strains were named A2G0016 (Δ*nfnAB*), A2G0017 (Δ*nfnB*), and A2G0018 (Δ*nfnA*) and were submitted to HPLC analysis after fermentation. Both the M1442 and the LL1328 strains have the same ethanol titers around 180 mM after 240 h of fermentation, which corresponds to 90% of the theoretical maximum in *T. saccharolyticum* (Fig. 2). The knockouts affected ethanol levels in different ways: while the Δ*nfnA* strain produced ethanol titers around 105 and 135 mM after 120 h or 240 h, respectively (about 52.5% and 67.5% of the theoretical maximum), the Δ*nfnB* strain reached ethanol titers of 43 and 62 mM after 120 h or 240 h, respectively, representing only 21.5% and 31% of the theoretical maximum (Fig. 2 and Fig. S1). The ethanol titers exhibited by this last strain were similar to the those of the double-knockout Δ*nfnAB*, which is around 35%, as previously seen (Fig. 2 and [6]). These results suggest that NfnB has some function independent of the NfnAB complex, while NfnA does not, which means that NfnA only functions as part of the NfnAB complex, whereas NfnB can function either by itself or as part of the NfnAB complex. In addition, our results point out that although the NfnAB complex is important for NADPH-FNOR activity (6), it is not responsible for all of the electron flux from ferredoxin to NADP^+^ in the ethanologenic strain.

In the past, our group also identified a new NADH-FNOR in *T. saccharolyticum*, the Tsac_1705 protein, whose deletion in a wild-type strain resulted in a 75% loss of NADH-FNOR activity, but did not alter the ethanol titer concentration (8). In order to investigate the role of this gene in an ethanologenic *T. saccharolyticum* strain, the *tsac_1705* gene was also deleted in the LL1328 strain, resulting in strain A2G0015. Again, the *tsac_1705* knockout did not modify the ethanol titer of *T. saccharolyticum* (Fig. 2), showing that this gene does not play a significant role in achieving high ethanol titers in the *T. saccharolyticum* ethanologenic strain.

Recently, another publication from our group has shown that hydrogenases from the *hyd* and *hfs* gene clusters enable high ethanol yield in a different organism, *T. thermosaccharolyticum*. A hypothesis of redox balance via hydrogen cycling has been suggested in which FNORs and hydrogenases, more specifically HydAB and HfsD, are essential for this trait (9). To further explore this, we deleted the *hydA* and *hfsD* hydrogenases in the *T. saccharolyticum* LL1328 strain as well, resulting in strains A2G0019 and A2G0020, respectively. *hydA* and *hfsD* knockouts resulted in ethanol titers of 55 and 40 mM, respectively, both after 120 h and 240 h, representing 27.5% and 20% of the theoretical maximum, respectively (Fig. 2 and Fig. S1). The double-deletion strain (A2G0021), in turn, resulted in 52 and 59 mM of ethanol at 120 h and 240 h, representing 26% and 29.5% of the theoretical maximum, respectively (Fig. 2 and Fig. S1). This large decrease in ethanol levels suggests that both genes are necessary to allow electron transfer from ferredoxin for high-yield ethanol production. To summarize: four genes are essential for high-yield ethanol production in *T. saccharolyticum*, namely, *nfnA*, *nfnB*, *hydA*, and *hfsD*.

The impact of the knockouts on molecular hydrogen production was also investigated through GC analysis. All the *nfn* deletions (Δ*nfnA,* Δ*nfnB,* and Δ*nfnAB*) resulted in increased hydrogen levels (2.3, 2.4, and 2.5 times higher than those of the LL1328 parental strain, respectively) (Fig. 2). These results are consistent with those of a previous work by our group that showed increased H_2_ levels for another *nfnAB* deletion strain (6). H_2_ production also increased when *nfnAB* was knocked out in the closely related organism, *Thermoanaerobacterium aotearoense* (20). As for the other mutants (Δ*tsac_1705,* Δ*hydA,* Δ*hfsD,* and Δ*hydA* Δ*hfsD*), there was no significant difference. It is possible that other hydrogenases from the same *hfs* and *hyd* clusters are compensating for the absence of *hfsD* and *hydA*.

In order to evaluate the effect of the mutations on the growth profile of the strains, we performed a growth analysis over a 96 h period. All mutant strains exhibited a reduced growth rate of more than 30% (Fig. 2 and Fig. S2), suggesting the presence of a redox imbalance, with the exception of Δ*nfnA* and Δ*tsac_1705* strains.

### Effect of knockouts on substrate consumption and fermentation products other than ethanol

**Fig 3:**
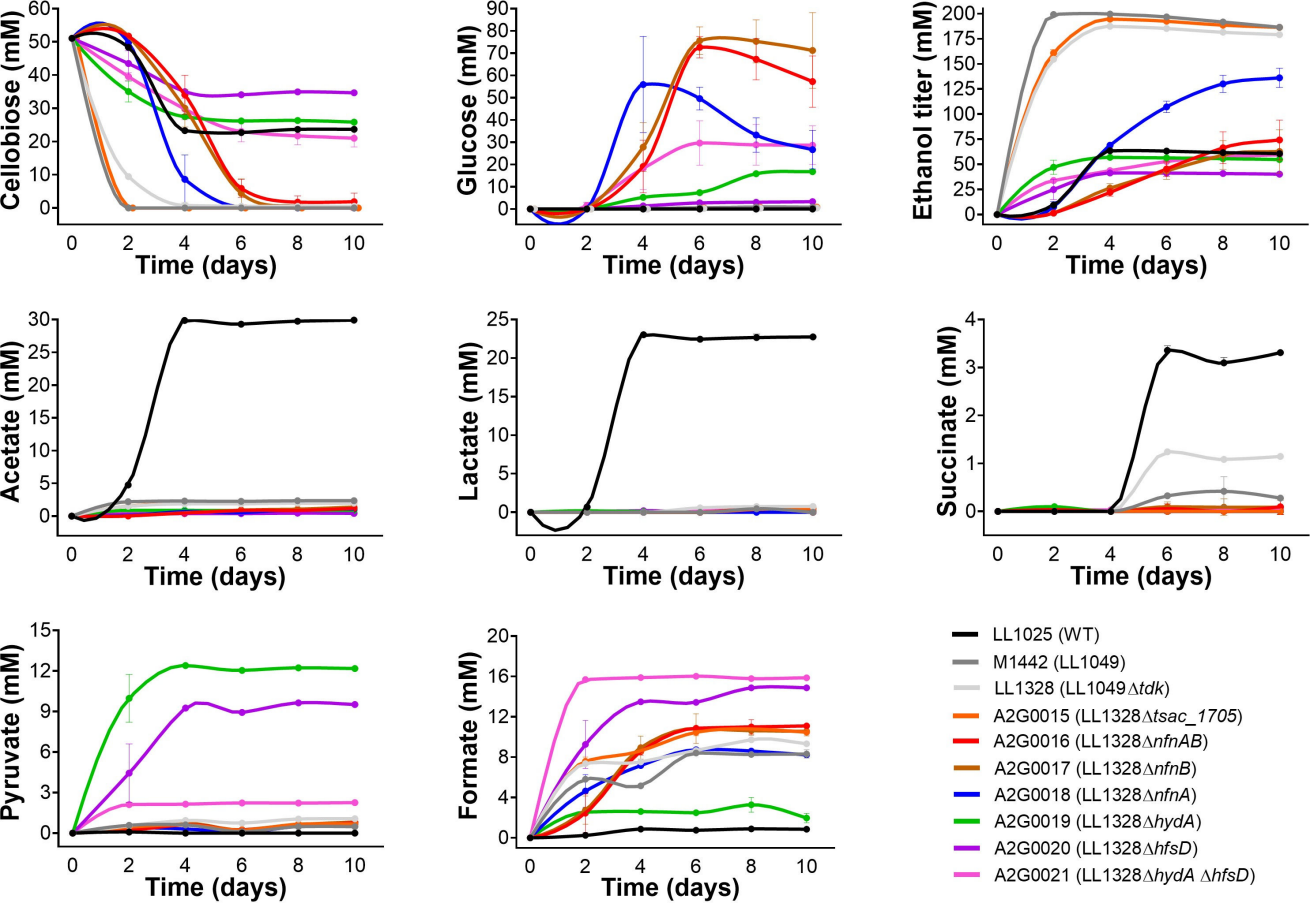
Substrate consumption and production of products other than ethanol for *T. saccharolyticum* knockout mutants. All the strains were cultivated on the MTC-6 defined medium containing 20 g/L of cellobiose for 10 days. Error bars represent one standard deviation with *n* ≥ 2 biological replicates. All fermentation data are reported in Table S2.

To assess the concentration of other fermentation metabolites, the fermentation profiles of all mutant strains were analyzed every 2 days during a 10-day incubation (Fig. 3). Strains M1442 (ethanologenic), LL1025 (wild-type), and LL1328 (ethanologenic) consumed all the 54 mM of cellobiose in the medium in 4 days or less, and almost no glucose was generated. On the other hand, strains Δ*nfnA,* Δ*nfnB,* and Δ*nfnAB* consumed almost all cellobiose after 6 days of fermentation with production of large amounts of glucose. And these high levels of glucose were maintained until day 10, especially in the *nfnB* and *nfnAB* knockout strains, showing that a large amount of the substrate is not consumed by these mutants. As for the Δ*hydA*, Δ*hfsD,* and Δ*hydA* Δ*hfsD* strains, cellobiose consumption ceased after 4 days of fermentation, with more than 50% of its initial concentration still present at the end of fermentation. However, while the *hfsD* knockout produced almost no glucose, *hydA* and the double *hydA hfsD* knockouts produced, respectively, 20 and 30 mM glucose at the end of the experiment.

As expected, all knockouts showed virtually no acetate and lactate production (Fig. 3) since they derive from the M1442 strain, which was generated by deleting several genes including *ldh* (lactate dehydrogenase) and *pta-ack* (phosphotransacetylase and acetate kinase) (1). They also produced virtually no succinate. Interestingly, all strains produce pyruvate, the final product of glycolysis (Fig. 1), but only Δ*hydA* and Δ*hfsD* mutants presented elevated levels of this metabolite. Levels of formate, an alternative product generated from pyruvate, varied among the strains, with strains with *hfsD* deleted exhibiting formate production almost twice that of other strains at the end of the experiment.

**Fig 4:**
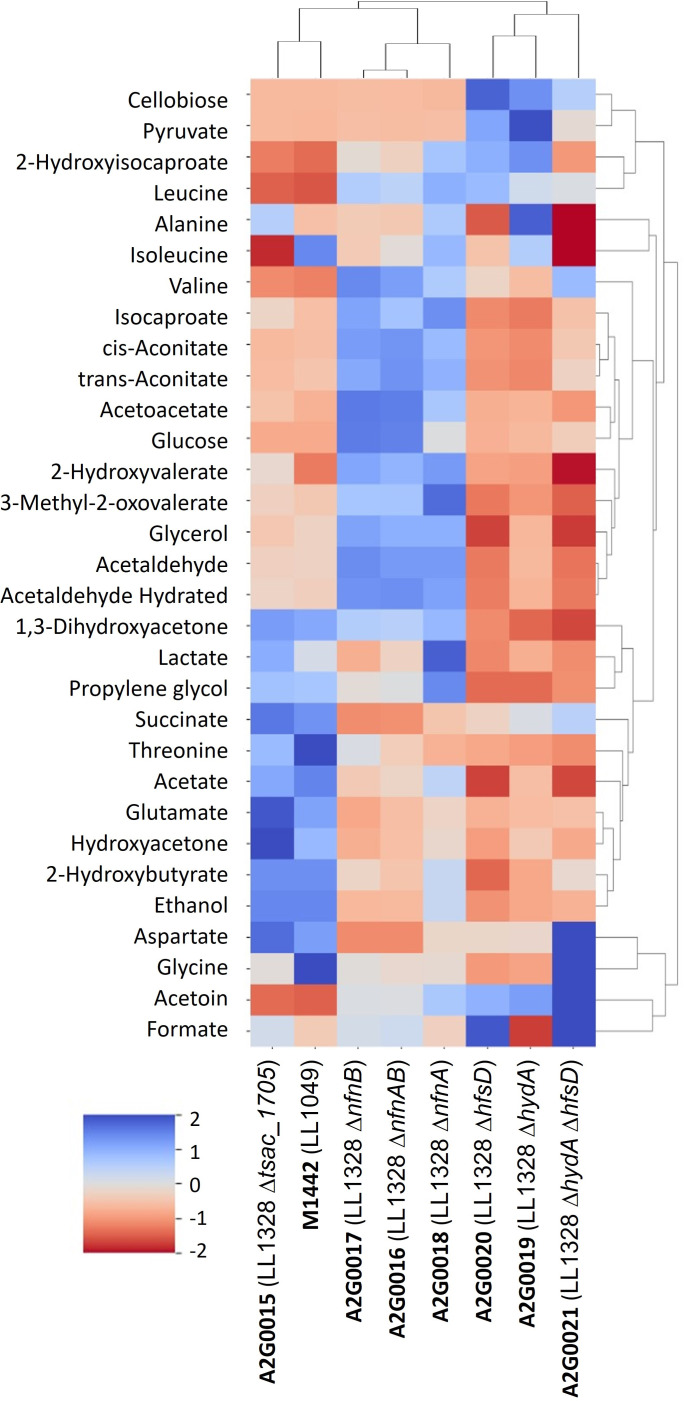
NMR analysis of the fermentation of *T. saccharolyticum* knockout mutants. All the strains were cultivated on the MTC-6 defined medium containing 20 g/L of cellobiose for 240 h. Samples were filtered and submitted to NMR analysis in duplicates. The values underwent Z-score normalization and were hierarchically clustered by Ward’s linkage method using seaborn clustermap (Python). All NMR data are reported in Table S4.

Since the carbon recovery of some strains remained low until the end of the experiment, we decided to submit the fermentation samples of the mutant strains to NMR analysis in order to identify and quantify possible metabolites and fermentation intermediates not quantified by HPLC. The strains and quantified metabolites were clustered and arranged in a heat map shown in Fig. 4. Unsurprisingly, *tsac_1705* knockout clustered with the M1442 ethanologenic strain, and both presented almost the same metabolic profile, with only a few differences, such as the concentration of the amino acids alanine and isoleucine. The *nfn* mutant strains also clustered with themselves, with Δ*nfnB* and Δ*nfnAB* showing very similar metabolic profiles. Notably, the three *nfn* mutants accumulated acetaldehyde, acetoacetate, glycerol, and aconitate. Since the last step of the ethanol production pathway (the conversion of acetaldehyde to ethanol) is NADPH-dependent (Fig. 1), the accumulation of acetaldehyde and the decrease in ethanol titers in *nfn* mutants are explained because the NfnAB complex is an important source of NADPH. Without the NADPH-FNOR activity in *nfn* knockouts, NADPH production is impaired, and consequently, there is less conversion of acetaldehyde to ethanol.

The hydrogenase mutants also cluster among themselves and, in general, have lower levels of fermentation products compared to the other strains, but higher levels of pyruvate and acetoin (Fig. 4), an intermediate of amino acid metabolism that helps prevent internal bacterial acidification due to pyruvate accumulation (21). Once again, it is clear that *hfsD* deletion leads to formate accumulation, an indication that loss of function of this hydrogenase blocks the ethanol production pathway in the conversion of pyruvate to acetyl-CoA (where pyruvate:ferredoxin oxidoreductase acts), favoring the conversion of pyruvate to formate by pyruvate-formate lyase (Fig. 1). Furthermore, amino acid metabolism is strongly impacted by the double deletion of *hydA* and *hfsD* since accumulation of aspartate and glycine and low abundance of alanine and isoleucine can be observed in the Δ*hydA* Δ*hfsD* double knockout (Fig. 4).

**Fig 5:**
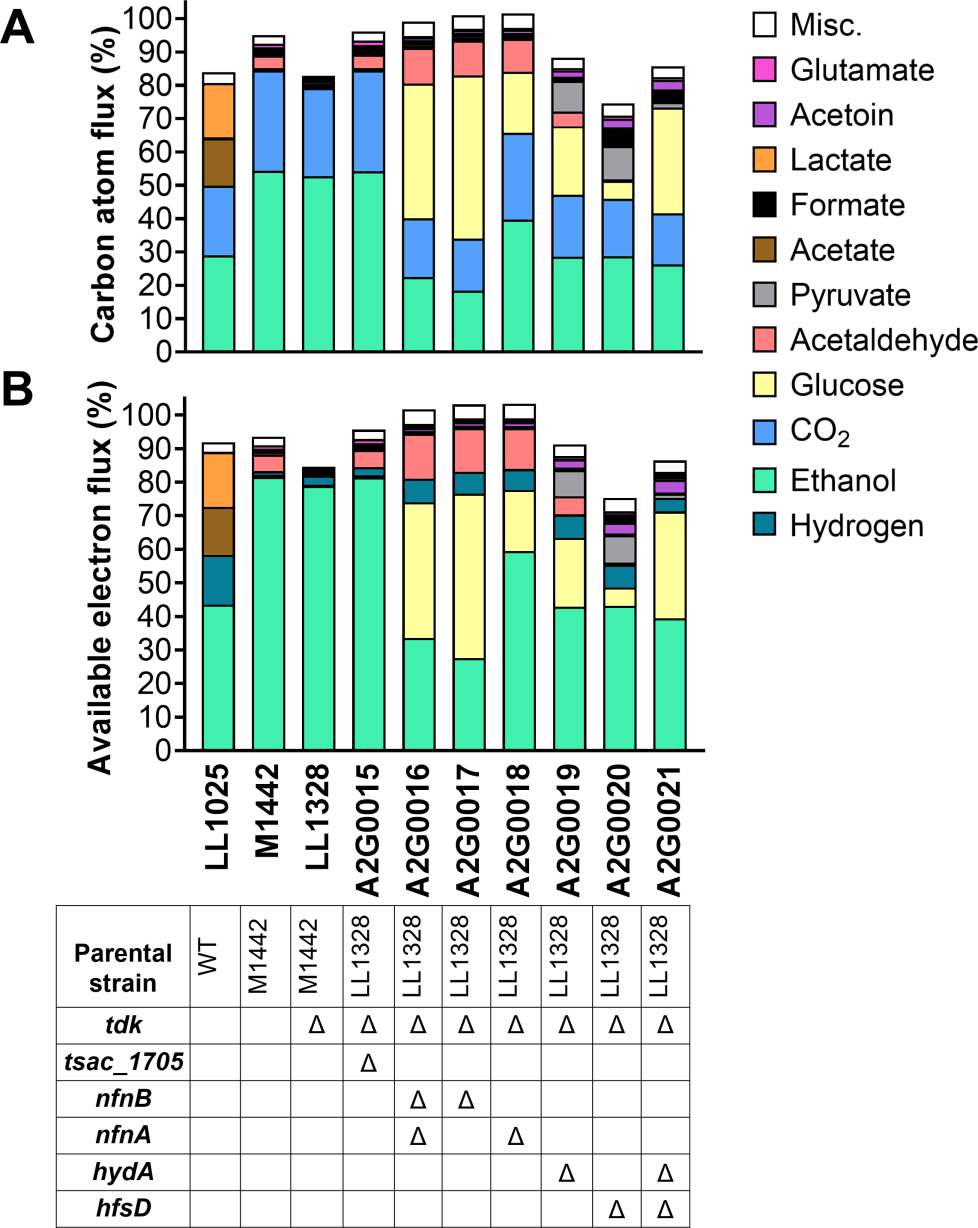
Carbon and available electron balances for engineered strains. For both carbon (**A**) and electron (**B**) flux, data for the top 10 compounds are presented individually, and the remaining compounds are lumped together as “Misc.” The genotype of each strain is shown in the table below the graphics, where blanks indicate the WT alleles and Δ indicates disruption of the gene by replacement with a *kan* marker. All fermentation analysis and material balance calculations are reported in Table S5.

Given the considerable differences between the mutant strains, we decided to perform a carbon and electron flux analysis using data from HPLC, NMR, and GC analyses, in order to understand the impacts of the mutations on carbon and electron balances during fermentation. Most of our strains exhibited >85% carbon recovery (Fig. 5), before accounting for cell mass. Previously, we have observed 3%–10% of carbon flux to cells (22, 23), suggesting that carbon balances are approximately closed within our experimental accuracy (95%–105%). Two exceptions are the WT (LL1025) and one ethanologenic strain (LL1328), where NMR measurements were not performed on fermentation products. Thus, in these strains, some of the unmeasured carbon is likely present in acetaldehyde, acetoin, and glutamate (and the CO_2_ associated with the formation of these compounds). One notable exception is strain A2G0020 (Δ*hfsD*), which has low carbon recovery (75%) despite the inclusion of NMR data. It is likely that the unknown carbon represents one or more unmeasured fermentation products; however, their identity remains a mystery.

### Secondary mutations in *T. saccharolyticum* knockout strains

The emergence of spontaneous mutations in bacteria is a very common secondary effect when a major disturbance occurs to the cell (24). In order to investigate possible compensatory mechanisms, the mutant strains were thus submitted for whole-genome sequencing to analyze secondary mutations associated with the gene deletions performed. Mutations in the DNA sequence at different locations across the genome were found in all the knockout strains, as listed in Table 2. The majority of them led to alterations in amino acid sequences. The Δ*nfnA* and Δ*hydA* Δ*hfsD* strains were the ones that accumulated the most mutations. Among the most relevant mutations for this work, a single-nucleotide variation in the gene sequence of the hydrogenase EchB stands out in the Δ*nfnA* strain due to its possible role in redox metabolism. The Ech hydrogenase is thought to couple hydrogen production with sodium or proton transport, with the B-subunit specifically involved in the transport step (25). However, its function in *T. saccharolyticum* is unknown, and previous work on deletion of this gene did not show a strong effect on either hydrogen production or the distribution of fermentation products (10).

**TABLE 2:** Secondary mutations in *T. saccharolyticum* mutant strains

Strain	Background	Locus	Protein product of the locus gene	Region	Type^*a*^	Mutation description	Frequency^*b*^
*∆tsac_1705*	M1442	*tsac_2162*	Pyruvate flavodoxin/ferredoxin oxidoreductase	2201725	SNV	G → T, Ala295Glu	99%
*∆tsac_1705*	M1442	*tsac_1725*	Protein serine/threonine phosphatase	1799018	SNV	A → T, Ala50Thr	99%
*∆nfnB*	M1442	*tsac_R0010*	tRNA-Thr	1042690	SNV	T → C	99%
*∆nfnB*	M1442	*tsac_1044*	ABC transporter	1077267	SNV	G → A, Arg229Lys	100%
*∆nfnA*	M1442	*tsac_0617*	CrcB-like protein	652770	SNV	C → A, Thr37Lys	95%
*∆nfnA*	M1442	*tsac_0676*	echB (PFAM: NADH:ubiquinone oxidoreductase)	715783	SNV	A → G, Thr185Ala	50%
*∆nfnA*	M1442	*tsac_1419*	ATP synthase subunit a	1492137	SNV	C → A, Ser178Arg	100%
*∆nfnA*	M1442	*tsac_1694*	Single-stranded-DNA-specific exonuclease RecJ CDS	1764492	SNV	A → G	100%
*∆nfnA*	M1442	*tsac_1725*	Protein serine/threonine phosphatase	1799917	SNV	G → A, Ala50Thr	100%
*∆nfnA*	M1442	*tsac_1917*	Sporulation transcriptional activator Spo0A	1974802	Deletion	Frameshift	95%
*∆nfnA*	M1442	*tsac_1987*	Abortive infection protein	2034016	SNV	T → C, Thr76Ala	100%
*∆nfnAB*	M1442	*tsac_0085*	Phosphoesterase PA-phosphatase-like protein	84540	SNV	G → A	100%
*∆hydA*	M1442	*tsac_0438*	UDP-N-acetylglucosamine 1-carboxyvinyltransferase	469474	SNV	T → C, Lys257Arg	100%
*∆hydA*	M1442	*tsac_0593*	Family 2 glycosyl transferase	623432	SNV	G → T, Arg70Ser	100%
*∆hydA*	M1442	*tsac_0794*	Hypothetical protein	831106	SNV	C → A, Gly185Val	100%
*∆hydA*	M1442	*tsac_0923*	Hypothetical protein	968497	SNV	T → C	100%
*∆hydA*	M1442	*tsac_1989*	Cysteine desulfurase	2035425	SNV	C → A, Val188Leu	100%
*∆hfsD*	M1442	*tsac_0438*	UDP-N-acetylglucosamine 1-carboxyvinyltransferase	469474	SNV	T → C, Lys257Arg	100%
*∆hfsD*	M1442	*tsac_0593*	Family 2 glycosyl transferase	623432	SNV	G → T, Arg70Ser	100%
*∆hfsD*	M1442	*tsac_0794*	Hypothetical protein	831106	SNV	C → A, Gly185Val	100%
*∆hfsD*	M1442	*tsac_0923*	Hypothetical protein	968497	SNV	T → C	100%
*∆hfsD*	M1442	*tsac_1989*	Cysteine desulfurase	2035425	SNV	C → A, Val188Leu	100%
*∆hydA ∆hfsD*	LL1328	*tsac_0131*	CheW protein	134796	Deletion	Frameshift	99%
*∆hydA ∆hfsD*	LL1328	*tsac_0182*	Transposase IS4 family protein CDS	192960	SNV	G → A, Asp88Asn	50%
*∆hydA ∆hfsD*	LL1328	*tsac_0310*	Hypothetical protein	327841	SNV	G → A	50%
*∆hydA ∆hfsD*	LL1328	*tsac_0902*	Hypothetical protein	945063	SNV	C → T, Pro177Ser	40%
*∆hydA ∆hfsD*	LL1328	*tsac_1205*	FAD-dependent oxidoreductase	1244407-1245422	Deletion		100%
*∆hydA ∆hfsD*	LL1328	*tsac_1206*	FAD-dependent pyridine nucleotide-disulfide oxidoreductase	1245654-1245851	Deletion		100%
*∆hydA ∆hfsD*	LL1328	*tsac_1551*	Hydrogenase hfsB	1627817	Deletion	Frameshift	90%
*∆hydA ∆hfsD*	LL1328	*tsac_1820*	4-hydroxy-3-methylbut-2-en-1-yl diphosphate synthase CDS	1880177	SNV	G → A, Arg268Lys	100%
*∆hydA ∆hfsD*	LL1328	*tsac_2615*	Redox-sensing transcriptional repressor Rex	2664101	Deletion	Frameshift	85%

^*a*^ SNV: single-nucleotide variation.

^*b*^ percentage of sequencing reads that contain mutation.

In the double-knockout Δ*hydA* Δ*hfsD*, spontaneous deletions in four different genes seemed particularly interesting. One mutation was a deletion of the genes *tsac_1205* and *tsac_1206*. These genes are part of an operon associated with glycerol production (26–28), and their deletion seems to have slightly decreased glycerol production (Fig. 4). Another mutation of interest was a frameshift mutation in the *hfsB* gene. Previously, we have seen this type of mutation in strains of *T. saccharolyticum* engineered for high-yield ethanol production (23). Our current understanding is that this mutation deregulates *hfsD*, although it does not explain the selective benefit for this mutation in an *hfsD* deletion strain. Finally, we observed a frameshift mutation in the *rex* gene. The *rex* gene senses the NADH/NAD^+^ ratio and regulates oxidoreductases, such as AdhE, which are responsible for maintenance of redox balance. Previously, we have shown that deletion of *rex* in *T. saccharolyticum* results in increased ethanol production by increased *adhE* expression (29).

## DISCUSSION

In this work, we set out to understand the role of different genes involved in electron flux in a *T. saccharolyticum* strain engineered for increased ethanol production. Electron flux is an important factor that can either limit or boost ethanol production in anaerobic thermophilic bacteria. Recently, using the *C. thermocellum* cell lysate, we found that carbon flux is strongly controlled by electron availability (30), and this can impact ethanol production. In fact, previous studies have shown that ethanol production can be increased by disrupting competing pathways for electron flux, such as hydrogen production (11, 31). Here, we showed that ethanol titer in *T. saccharolyticum* decreases when any of the following genes are absent: *nfnB*, *hydA*, *hfsD*, and *nfnA* to a lesser extent.

For high-yield ethanol production, the generation of reduced cofactors must exactly balance their consumption. Recent investigations into the AdhE enzyme in engineered ethanologenic strains of *T. saccharolyticum* have resolved the question of reduced cofactor consumption [manuscript JB00015-25 in press at the Journal of Bacteriology]. The conversion of acetyl-CoA to acetaldehyde uses NADH as a cofactor, and the conversion of acetaldehyde to ethanol uses NADPH (Fig. 1). The current work therefore focuses on how these cofactors are generated.

One key question is the stoichiometry of the NFN reaction. The NFN reaction couples an exergonic transfer of electrons from Fd_red_ to NADP^+^, which is coupled to an endergonic transhydrogenation reaction (NADH → NADPH). If those reactions are obligately coupled, we would need an additional NADH-linked FNOR reaction to balance stoichiometry with the known cofactor consumption. However, if those reactions are not obligately coupled, no additional NADH-linked FNOR reaction is necessary.

The initial characterization of the NFN reaction suggested that the reactions were obligately coupled (7), and this was supported by additional structural and biochemical evidence based on the study of NfnAB proteins from other organisms (25, 32). Despite that, there has also been some evidence that these reactions are not obligately coupled. For example, the NfnL from *Pyrococcus furiosus* (corresponding to NfnB from *T. saccharolyticum*) was shown to allow electron transfer from Fd_red_ to NADP^+^ even in the absence of NfnS (corresponding to NfnA from *T. saccharolyticum*) (33). In fact, the electron transfer activity from Fd_red_ to NADP^+^ occurs mainly in the absence of NfnS (NfnA) and is only minimal when the entire complex (NfnSL/NfnAB) is present. Another example is the Cac_0764 enzyme, which is similar to NfnB at the amino acid level (41.7% identity) and has also been shown to mediate electron transfer from Fd_red_ to NADP^+^ (34). Our results appear to show that NfnB can function independently of NfnA (Fig. 2 and 3). This is based on the observation that individual deletion of *nfnA* and *nfnB* genes results in distinct fermentation phenotypes. The deletion of *nfnB* has a similar phenotype to that of the *nfnAB* deletion, suggesting that *nfnA* only functions in the context of the NfnAB protein complex. However, the *nfnA* deletion (i.e., where *nfnB* is present in the absence of *nfnA*) exhibits increased ethanol yield (Fig. 2). Despite this, it seems like independent activity of the NfnB subunit may have low physiological relevance due to the low rate of ethanol production allowed by this activity (Fig. 3). In addition, the accumulation of acetaldehyde in this strain (Fig. 4) further suggests that the rate at which NfnB is regenerating NADPH is limiting ethanol production.

Assuming the NFN reaction is obligately coupled under most physiological conditions, we need a separate ferredoxin NAD^+^ oxidoreductase to enable high-yield ethanol production. Previously, we have suggested that this is due to the activity of the *tsac_1705* gene. However, here we provide definitive evidence that this gene does not play an important role in electron transfer for ethanol production. This is based on our observation that deletion of this gene does not affect ethanol yield (Fig. 2) or the rate of ethanol production (Fig. 3).

An alternative possibility for this reaction is the combination of multiple hydrogenases via a process called hydrogen cycling. This has been recently proposed to work in *T. thermosaccharolyticum* as follows (9). The Hyd reaction (Equation 3), mediated by the *hfsD* gene, proceeds in the forward direction to generate hydrogen from reduced ferredoxin. This hydrogen is then taken up by the reversible bifurcating hydrogenase (Bif-Hyd, Equation 4), mediated by the *hydABCD* gene cluster. These reactions can be combined in a 2:1 ratio to give a net reaction identical to the NADH-producing FNOR reaction (Equation 2):


(Eq. 3) $$2\;Fd_{red}^{1-}+2\;H^{+}\overset{}{\to }2\;Fd_{ox}+1\;H_{2}$$

(Eq. 4) $$2\;H_{2}+NAD^{+}+2\;Fd_{ox}\overset{}{↔}3\;H^{+}+NADH+2\;Fd_{red}^{1-}$$

This reaction appears to be functioning in *T. saccharolyticum* as well. The similarity of the fermentation phenotypes for strains where either *hydA*, *hfsD*, or both *hydA* and *hfsD* are deleted has been supporting evidence (Fig. 2 and 4). However, additional biochemical characterization will be required to definitively determine this mechanism of electron transfer. The presence of hydrogen cycling may explain the high ethanol yields observed in a strain of *T. saccharolyticum* engineered for ethanol production that lacks *nfnAB* (i.e., strain LL1145) (6). In this strain, both of the final steps of ethanol production from acetyl-CoA use NADH as the electron donor, avoiding the need for the NFN reaction.

This understanding of the relative roles of NfnAB (for NADPH production) and the HfsD and HydA hydrogenases (for NADH production via hydrogen cycling) also sheds light on the redistribution of fermentation products when these genes are deleted. Disruption of NADPH regeneration (via *nfnA*, *nfnB*, or *nfnAB* deletion) resulted in an excess of NADH and lack of NADPH. This allowed the NADH-linked ALDH reaction to proceed rapidly, producing acetaldehyde (Fig. 4), but this acetaldehyde accumulated due to a lack of NADPH needed for its further conversion to ethanol. By contrast, disruption of the hydrogenases thought to be involved in hydrogen cycling resulted in a lack of NADH. This limited the ethanol production pathway at the NADH-linked ALDH reaction, causing an accumulation of upstream metabolites including pyruvate and acetoin (Fig. 4). Almost all of the strains with disruptions in electron transfer pathways accumulated glucose (Fig. 3 and 5). Our current understanding of cellobiose metabolism in this organism does not provide any obvious explanation for this conversion beyond the trivial one: that this is a reaction that can occur when other metabolic reactions are blocked due to redox imbalances.

One other interesting question raised by this work is why *T. saccharolyticum* developed a complex system of electron transfer requiring at least four genes (*hfsB*, *hydA*, *nfnA*, and *nfnB*), when a simpler system (*nfnB* only) seems to be biologically possible? We do not have a definitive answer to this question but suspect that this complex pathway may represent a “good-enough” approach for the organism, and the relative ease of generating loss-of-function mutations (vs gain-of-function mutations) may have biased the evolutionary trajectory of the organism to favor this approach.

In conclusion, in this work, we have investigated the role of different enzymes in the generation of reduced cofactors for the ethanol production pathway of *T. saccharolyticum*. We provided evidence that four genes are essential for high-yield ethanol production in this organism: *nfnA*, *nfnB*, *hydA*, and *hfsD*. Our data suggested that NfnB may function independently of NfnA; however, the independent activity of NfnB is not sufficient to explain the rate of ethanol production observed in the engineered ethanologen strain, M1442.

Instead, the NFN reaction appears to work in combination with the Hfs and Hyd enzymes. Given the importance of HfsD and HydA for high-yield ethanol production in *T. saccharolyticum*, the hydrogen cycling hypothesis is an open field for future investigation. Its biophysical mechanism, regulation, and extent of coupling (i.e., which portion of hydrogen is channeled and which portion escapes) are some key points that still need to be elucidated. Such findings may suggest the applicability of transferring this ethanol production pathway to other organisms, with potential implications for industrial biofuel production.

## SUPPLEMENTAL MATERIAL

Figure S1 jb.00107-25-s0001.tif Ethanol titers of *T. saccharolyticum knockout mutants.*

Figure S2 jb.00107-25-s0002.tif Growth curves of the strains.

Table S1 jb.00107-25-s0003.xlsx Primers used for *T. saccharolyticum transformation and validation.*

Table S2 jb.00107-25-s0004.xlsx Batch fermentation data measurements.

Table S3 jb.00107-25-s0005.xlsx Molecular hydrogen measurements.

Table S4 jb.00107-25-s0006.xlsx Nuclear magnetic resonance data.

Table S5 jb.00107-25-s0007.xlsx Material balance calculations.

ASM does not own the copyrights to Supplemental Material that may be linked to, or accessed through, an article. The authors have granted ASM a non-exclusive, world-wide license to publish the Supplemental Material files. Please contact the corresponding author directly for reuse.
