# Supplementary figures and images for: Identification of electron transfer enzymes in Thermoanaerobacterium saccharolyticum
Source: J Bacteriol. 2025 Jun 6;207(7):e00107-25. doi: 10.1128/jb.00107-25 (PMC12288451; doi:10.1128/jb.00107-25)

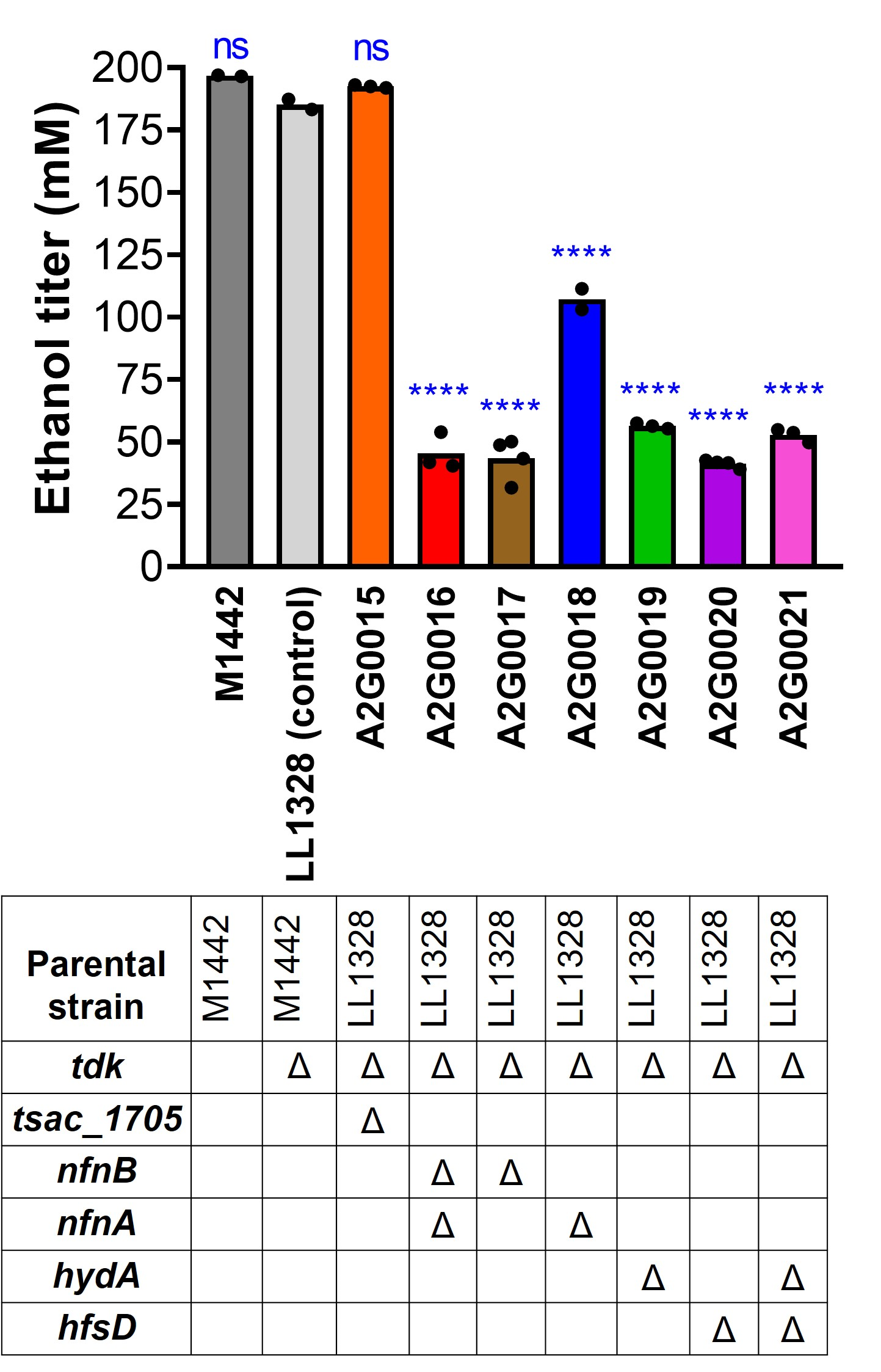

Supplement: Figure S1 — Ethanol titers of T. saccharolyticum knockout mutants. [file jb.00107-25-s0001.tif]

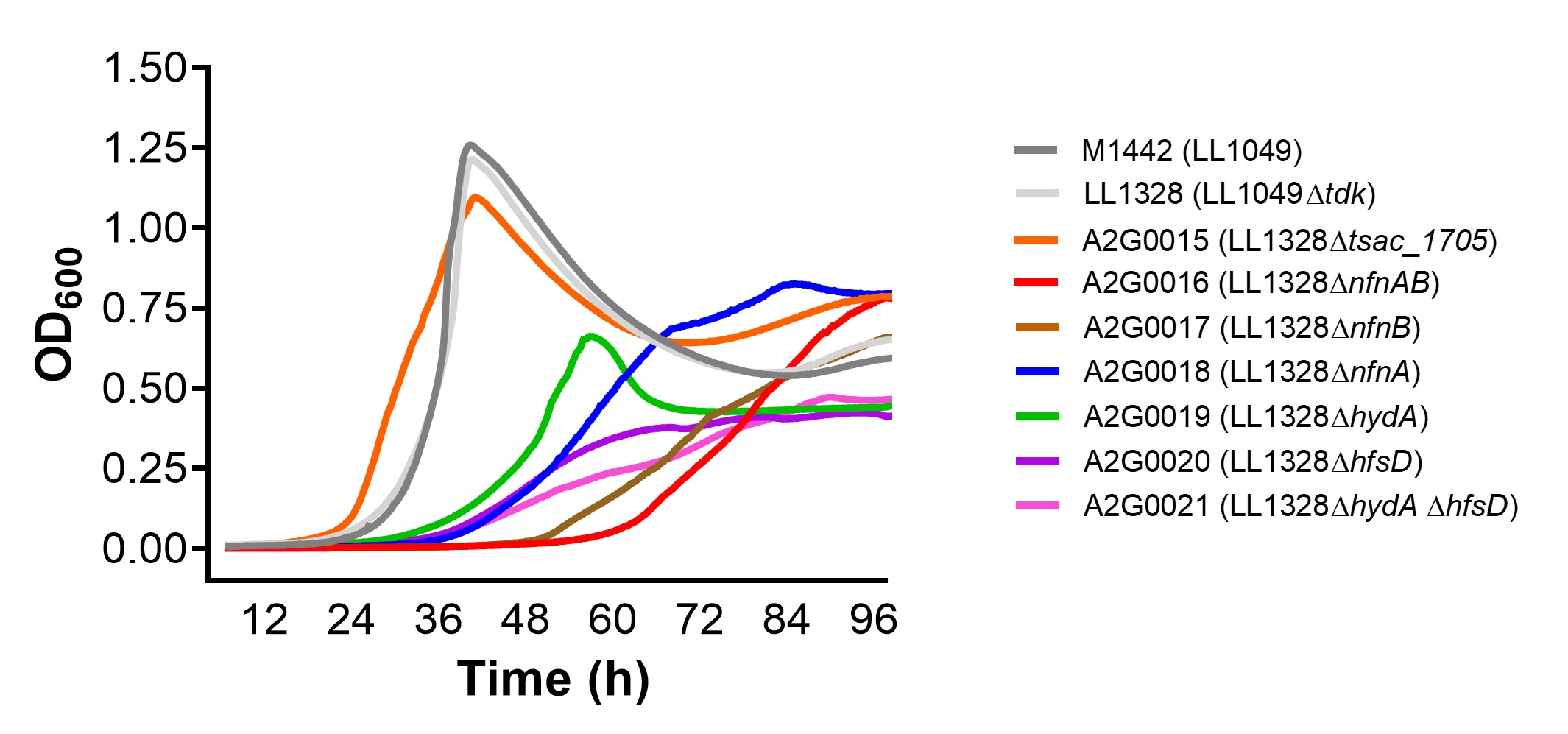

Supplement: Figure S2 — Growth curves of the strains. [file jb.00107-25-s0002.tif]
